# Brain CT scan results in Epileptic patients referred to neurologic clinics in Kermanshah in 1375-86

**Published:** 2012-11

**Authors:** Daryoosh Afshari, Sahar Moradi, Hanieh Shahande, Katayoon Bavandpour, Elham Gilvary, Marzieh Allaei

**Affiliations:** ^*a*^Kermanshah University of Medical Sciences, Kermanshah, Iran.; ^*b*^Vice Chancellery for Research and Technology, Kermanshah University of Medical Sciences, Kermanshah, Iran.

## Abstract

**Background:**

The analysis of the brain CT Scan results in patients with epilepsy referred to the neurology clinic of Kermanshah during 1996-2007. Epilepsy is a transient disorder of the nervous system due to sudden discharge of brain neurons. The sudden and abnormal neuronal discharge may lead to consciousness level reduction, changes in perception or mental dysfunction of seizure movements, sensorineural disorders or a combination of these symptoms.

**Methods:**

This was a descriptive-cross sectional study. Patients with epilepsy referred to the neurology clinic in Kermanshah (Iran) who were studied by Brain CT scan during 1996-2007 were investigated. After confirming the epilepsy of patients by neurosurgery experts and removal of the seizure of consciousness loss due to other causes such as syncope, 931 patients were enrolled in our study.

**Results:**

Among 931 patients, only 905 patients had undergone Brain CT of which 464 were female and the rest were male. 325 patients suffered from focal motor epilepsy, patients referred with temporal epilepsy, 473 patients with Grand Mal epilepsy, 12 patients with absence epilepsy, 54 patients with myoclonic epilepsy, and 7 patients suffered from other types of epilepsy. The greatest Brain CT disorder was related to focal epilepsy. Among 125 patients with abnormal Brain CT, the greatest disorder was associated with cerebrovascular disorders (58 cases). In this study, the age range of samples was 1-89 years old. The lowest disorder was found in the age group of 10-19 and 20-39 years old (8.7% and 12.4%, respectively). The greatest disorder was observed in patients over 60 years old (38.3%). It should be noted that no Brain CT disorder was found in patients with youth myoclonic and absence epilepsy.

**Conclusions:**

The results of the present study can be helpful for proper use of brain imaging in patients with epilepsy.

**Keywords:**

Epilepsy, Brain CT, Neurology Clinic, Mayoclonic epilepsy


					Volume 4, Suppl. 1 Nov 2012
Publisher: Kermanshah University of Medical Sciences
URL: http://www.jivresearch.org
Copyright:
Congress of Iranian Neurosurgeons, 14-16 November 2012, Kermanshah, Iran
Paper No. 77
Brain CT scan results in Epileptic patients referred to
neurologic clinics in Kermanshah in 1375-86
Daryoosh Afshari a
, Sahar Moradi b,*
, Hanieh Shahande b
, Katayoon Bavandpour b
, Elham Gilvary a
,
Marzieh Allaei b
a
Kermanshah University of Medical Sciences, Kermanshah, Iran.
b
Vice Chancellery for Research and Technology, Kermanshah university of Medical Sciences, Kermanshah, Iran.
Abstract:
Background: The analysis of the brain CT Scan results in patients with epilepsy referred to the neurology clinic of
Kermanshah during 1996-2007. Epilepsy is a transient disorder of the nervous system due to sudden discharge of
brain neurons. The sudden and abnormal neuronal discharge may lead to consciousness level reduction, changes in
perception or mental dysfunction of seizure movements, sensorineural disorders or a combination of these symptoms.
Methods: This was a descriptive-cross sectional study. Patients with epilepsy referred to the neurology clinic in
Kermanshah (Iran) who were studied by Brain CT scan during 1996-2007 were investigated. After confirming the
epilepsy of patients by neurosurgery experts and removal of the seizure of consciousness loss due to other causes
such as syncope, 931 patients were enrolled in our study.
Results: Among 931 patients, only 905 patients had undergone Brain CT of which 464 were female and the rest
were male. 325 patients suffered from focal motor epilepsy, patients referred with temporal epilepsy, 473 patients
with Grand Mal epilepsy, 12 patients with absence epilepsy, 54 patients with myoclonic epilepsy, and 7 patients
suffered from other types of epilepsy. The greatest Brain CT disorder was related to focal epilepsy. Among 125
patients with abnormal Brain CT, the greatest disorder was associated with cerebrovascular disorders (58 cases). In
this study, the age range of samples was 1-89 years old. The lowest disorder was found in the age group of 10-19
and 20-39 years old (8.7% and 12.4%, respectively). The greatest disorder was observed in patients over 60 years
old (38.3%). It should be noted that no Brain CT disorder was found in patients with youth myoclonic and absence
epilepsy.
Conclusions: The results of the present study can be helpful for proper use of brain imaging in patients with
epilepsy.
Keywords:
Epilepsy, Brain CT, Neurology Clinic, Mayoclonic epilepsy
* Corresponding Author at:
Sahar Moradi: Vice Chancellery for Research and Technology, Kermanshah University of Medical Sciences, Kermanshah, Iran. Tel: 0831-8393159,
Email: saharmoradi_81@yahoo.com, (Moradi S.).

				

